# Brain Region-Specific Differences in Amyloid-β Plaque Composition in 5XFAD Mice

**DOI:** 10.3390/life13041053

**Published:** 2023-04-20

**Authors:** Angelika Sabine Bader, Marius-Uwe Gnädig, Merle Fricke, Luca Büschgens, Lena Josefine Berger, Hans-Wolfgang Klafki, Thomas Meyer, Olaf Jahn, Sascha Weggen, Oliver Wirths

**Affiliations:** 1 Department of Psychiatry and Psychotherapy, University Medical Center (UMG), Georg-August-University, 37075 Göttingen, Germany; 2 Department of Psychosomatic Medicine and Psychotherapy, University Medical Center (UMG), Georg-August-University, 37075 Göttingen, Germany; 3 Neuroproteomics Group, Department of Molecular Neurobiology, Max Planck Institute for Multidisciplinary Sciences, 37075 Göttingen, Germany; 4 Department of Neuropathology, Heinrich-Heine-University, 40225 Düsseldorf, Germany

**Keywords:** Alzheimer’s disease, amyloid, Abeta, 5XFAD, transgenic mice, plaque load, amino-terminal truncation, immunohistochemistry

## Abstract

Senile plaques consisting of amyloid-beta (Aβ) peptides are a major pathological hallmark of Alzheimer’s disease (AD). Aβ peptides are heterogeneous regarding the exact length of their amino- and carboxy-termini. Aβ1-40 and Aβ1-42 are often considered to represent canonical “full-length” Aβ species. Using immunohistochemistry, we analyzed the distribution of Aβ1-x, Aβx-42 and Aβ4-x species in amyloid deposits in the subiculum, hippocampus and cortex in 5XFAD mice during aging. Overall plaque load increased in all three brain regions, with the subiculum being the area with the strongest relative plaque coverage. In the subiculum, but not in the other brain regions, the Aβ1-x load peaked at an age of five months and decreased thereafter. In contrast, the density of plaques positive for N-terminally truncated Aβ4-x species increased continuously over time. We hypothesize that ongoing plaque remodeling takes place, leading to a conversion of deposited Aβ1-x peptides into Aβ4-x peptides in brain regions with a high Aβ plaque burden.

## 1. Introduction

Alzheimer’s disease (AD) is a progressive neurodegenerative disorder characterized by two major neuropathological hallmarks: intracellular neurofibrillary tangles consisting of hyperphosphorylated protein tau [1] and extracellular plaques, mainly composed of amyloid-beta (Aβ) peptides [2,3]. In addition to the vast majority of sporadic late-onset cases (SAD), early-onset familial cases (FAD) with autosomal-dominant inheritance have been described [4]. In most FAD cases, mutations in the amyloid precursor protein (APP) or the presenilin (PSEN1/PSEN2) genes affecting Aβ peptide metabolism have been identified, which is regarded as support for the hypothesis of Aβ having a pivotal role in AD [5,6]. Aβ peptides are physiological products of normal cellular metabolism and are formed via proteolytic processing of the large transmembrane APP protein by β-secretase and γ-secretase, usually resulting in the formation of Aβ peptides starting with aspartic acid residue at position 1 and with a length of 37–42 amino acids [7,8,9]. The most abundant form that is produced is Aβ1–40. While the physiological role of Aβ is yet to be fully discovered, in vitro studies indicate a possible role in homeostatic synaptic plasticity [10]. Depletion of Aβ led to neuronal death, which could be rescued by the application of Aβ at a physiological concentration in rat primary cortical neuronal cultures [11]. Furthermore, the picomolar concentration of synthetic Aβ1-42 peptides increased long-term potentiation in mouse hippocampal slices, while nanomolar concentrations led to its decrease [12]. In AD brains, Aβ peptides with varying C-termini accumulate in amyloid plaques and the vasculature [9], and it appears that the preferential deposition of Aβ42 in plaques is accompanied by a measurable decrease in soluble Aβ42 in cerebrospinal fluid (CSF), and also of the CSF ratio between Aβ42 and Aβ40. The latter can serve as a reliable neurochemical biomarker of amyloid pathology in AD [13]. Extracellular plaques are either characterized by a dense core region or a more diffuse pattern [14,15]. Plaques with a dense core region consist mostly of fibrillar Aβ peptides. Hyperphosphorylated tau protein as well as activated microglia and reactive astrocytes are co-located in those plaques. These cored plaques are defined as the mature form and are only found in later phases of amyloid pathology, while diffuse plaques are defined as more immature and are present during all phases of the pathology in AD patients as well as in many aged non-demented individuals [14]. However, even though they have already been described by Alois Alzheimer [16], the correlation between extracellular plaques and cognitive decline is weak [17]. In addition to “full-length” Aβ peptides, numerous other isoforms are found which are either truncated at the N- or C-terminus or show other post-translational modifications [3,9,18,19]. Along with “full-length” Aβ peptides, Aβ4-42 peptides starting with the phenylalanine residue at position 4 have been identified to represent a highly abundant isoform in the brains of FAD and SAD patients as well as in non-demented controls with amyloid pathology in different brain regions [3,20]. Heterogeneity of Aβ peptides, especially N-terminal truncated isoforms, were reported in AD patients as early as 1985 [3], and the identification of potential candidate enzymes being involved in the generation of such species from either APP or “full-length” Aβ [21,22] fueled current research interest. Amino-terminal truncated Aβ species display a higher probability to aggregate and oligomerize due to the changed kinetics following the loss of hydrophilic amino acids [23,24,25,26]. These truncated Aβ peptide variants are also found in transgenic AD mouse models including the 5XFAD model [22,27]. While, depending on the underlying mutations in APP and PSEN genes, increased Aβ levels in FAD cases may be associated with increased Aβ production, a disequilibrium between production and clearance mechanisms is regarded as a main contributor to Aβ accumulation in sporadic AD cases [28]. A variety of candidate proteases involved in brain Aβ degradation, such as neprilysin (NEP), insulin-degrading enzyme (IDE) or endothelin-converting enzyme (ECE) have been described in recent years [29]. In addition, the transport of soluble Aβ across the blood-brain barrier via Aβ transporters such as low density lipoprotein receptor-related protein 1 (LRP1) has been implicated in Aβ homeostasis [30].

5XFAD mice express human APP695 with the Swedish, Florida and London mutation as well as human PSEN1, the active site of γ-secretase, carrying the M146L and L286V mutation under the control of the murine Thy1-promotor [31,32]. Due to the Swedish mutation, the affinity of APP to β-secretase and, in return, the full-length Aβ peptide formation [33] is increased in 5XFAD mice. Being mainly a model for familial AD, these mice show first intraneuronal Aβ accumulation six weeks after birth [31,34]. Prominent Aβ peptide plaque pathology starts at an age of two to four months [31,32,35], with most plaques being located in the subiculum of the hippocampus and cortical layer V [31,32]. 5XFAD mice display reduced anxiety behavior, impaired motor control and neuroinflammation [31,32,36]. Furthermore, diffuse and neuritic plaques are also found in the brains of aged, non-demented human individuals [37,38]. 

In this study, we have investigated amyloid plaque deposition in the cortex, hippocampus and subiculum from two to ten months old 5XFAD mice by immunohistochemistry using a panel of antibodies differentiating between Aβx-42, Aβ1-x and Aβ4-x peptide variants. In this way, the amount and distribution of the different Aβ isoforms were investigated in an age-dependent fashion. The areas covered by either Aβx-42 or Aβ4-x positive plaques increased in all three brain regions over time. In the subiculum, but not in the hippocampus and cortex, the deposition of Aβ1-x followed a strikingly different time course than Aβx-42 and Aβ4-42. In this region, the Aβ1-x plaque load in the subiculum peaked at an age of five months and decreased afterward, suggesting plaque remodeling. 

## 2. Materials and Methods

### 2.1. Antibodies

Fluorescence staining was performed with the following monoclonal antibodies: 82E1 (mouse monoclonal Aβ1-x, 0.1 µg/mL, IBL, Hamburg, Germany), 18H6 (mouse monoclonal Aβ4-x, 1.9 µg/mL, [26], generous gift from Dr. J. Ghiso) and D3E10 (rabbit monoclonal Aβx-42, 1:500, Cell Signaling Technology, Frankfurt am Main, Germany). In addition, the novel rabbit polyclonal antibody 58-1 (Aβ4-x, 2 µg/mL; see Supplementary Materials for details) was applied together with 82E1 (0.2 µg/mL) for double staining.

### 2.2. Transgenic Mice

The generation of the 5XFAD mouse model (Tg6799) was described previously [31]. Mice were back-crossed to C57BL/6J wild-type mice (Jackson Laboratory, Bar Harbor, ME, USA) for more than 10 generations and maintained as a heterozygous transgenic line on a C57BL/6J background. Only male mice were included in the study (*n* = 5–7 per time point). Animals were housed in standard cages in a 12 h/12 h light-dark cycle. Food and water were available ad libitum. Animal experiments were performed according to the guidelines of the German animal protection law.

### 2.3. Immunohistochemistry on Paraffin Sections

Immunohistochemistry was performed on 4 µm thick sagittal paraffin mouse brain sections (bregma 0.96–1.20 mm). Sample processing was done as described previously [39]. In brief, paraffin was removed using Roticlear (Carl ROTH, Karlsruhe, Germany) and sections were rehydrated with an ascending ethanol series. Next, heat-induced antigen retrieval was carried out by boiling sections in 0.01 M citrate buffer (pH 6.0). Aβ epitopes were further exposed with an additional treatment with 88% formic acid. Sections were blocked in 4% skim milk in 0.01 M phosphate-buffered saline (PBS) with 10% fetal calf serum (FCS) for 1 h at room temperature (RT). Primary antibodies were diluted (dilution stated above) in 0.01 M PBS with 10% FCS and incubated overnight at RT in a humid chamber. Secondary antibody treatment was performed by incubation with the following fluorescent-labeled antibodies: AlexaFluor goat-anti-rabbit-594 (Invitrogen, Carlsbad, CA, USA), DyLight goat-anti-rabbit-550 (Thermo Fisher Scientific, Waltham, MA, USA) and AlexaFluor goat-anti-mouse-488 (Invitrogen) for 1.5 h at 37 °C. In addition, cell nuclei were stained using 4′,6-diamidin-2-phenylindol (DAPI, Sigma-Aldrich, Darmstadt, Germany).

### 2.4. Quantification of Aβ Load

Images were acquired using a Nikon TiE microscope equipped with a cooled DS-Qi2 camera and a motorized stage to construct large-scale images (Nikon, Tokyo, Japan) and analyzed using NIS Element imaging software (Nikon, Tokyo, Japan). Large-scale images covering the regions of interest were captured with 200× magnification and the ImageJ software package (V1.41, NIH, Bethesda, MD, USA) was used to generate binarized 8-bit black and white images. Subiculum, hippocampus (excluding subiculum) and cortex were delineated and a fixed intensity threshold was applied. Measurements were performed for the percentage area covered by extracellular amyloid plaques per region. Three sections per animal were averaged, which were at least 30 µm apart from each other.

### 2.5. Statistical Analysis

Means are presented with standard deviation (SD). Calculations and graphical presentation were prepared in GraphPad Prism version 9 for Windows (GraphPad Software, San Diego, CA, USA).

## 3. Results

**Figure 1:**
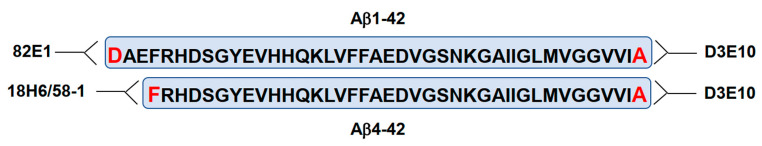
Amino acid sequences and numbering of Aβ1-42 and Aβ4-42 variants. Antibodies 82E1 and 18H6 or 58-1 detect the free N-terminus of full-length Aβ (Aβ1-x) or N-terminally truncated Aβ4-x irrespective of the C-terminus. Antibody D3E10 detects Aβ42 peptide variants with different N-termini (Aβx-42).

The relative area covered by plaques consisting of different Aβ species was measured by immunohistochemistry in the subiculum, the entire hippocampus and the cortex. We employed different antibodies, detecting either the free aspartic acid residue of full-length Aβ (Aβ1-x; 82E1), the free phenylalanine residue of N-terminally truncated Aβ4-x (18H6) or the free carboxy-terminal alanine residue of Aβ42, irrespective of the N-terminus (Aβx-42; D3E10) (Figure 1).

**Figure 2:**
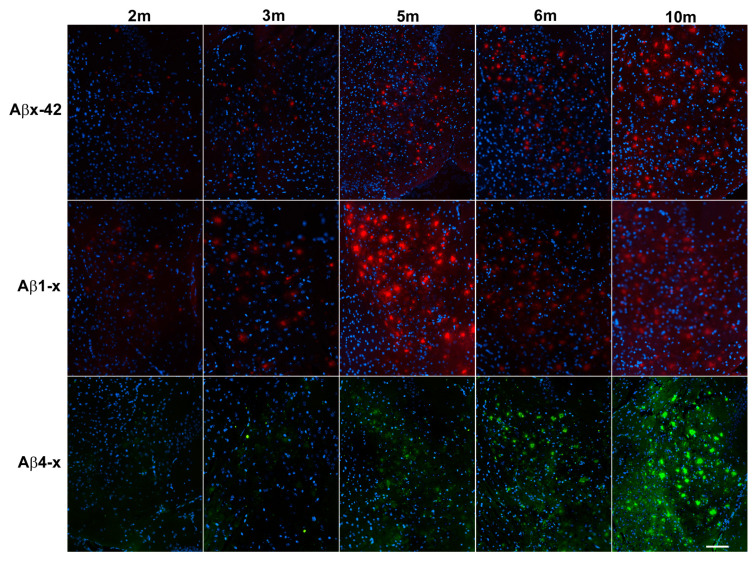
Representative images illustrating the plaque load for different Aβ species in the subiculum during aging. Aβx-42 staining (D3E10), Aβ1-x plaque pathology (antibody 82E1), and Aβ4-x plaque load (antibody 18H6). Scale bar: 250 µm.

**Figure 3:**
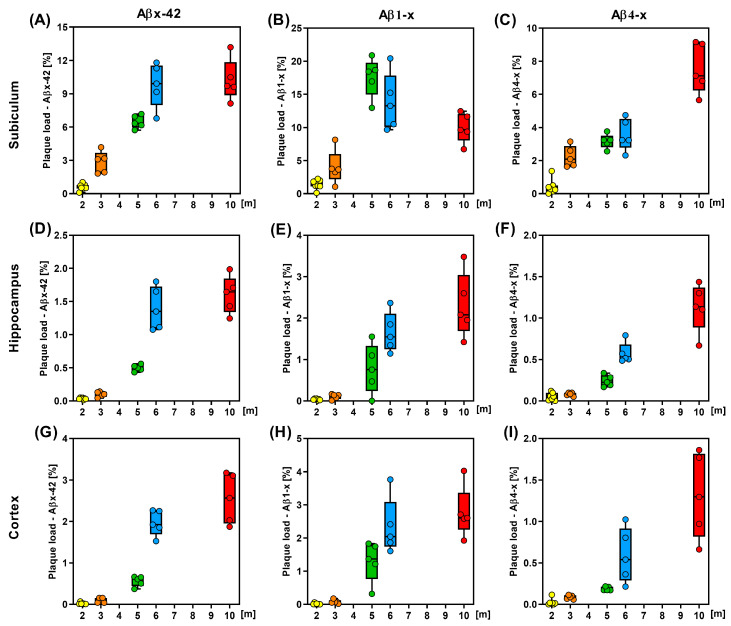
Quantitative analysis of the plaque load of different Aβ species. Aβx-42, (**A**,**D**,**G**); Aβ1-x, (**B**,**E**,**H**); Aβ4-x, (**C**,**F**,**I**) in male 5XFAD mice in the subiculum (**A**–**C**), hippocampus (**D**–**F**) and cortex (**G**–**I**) during aging (*n* = 7 (2 m), *n* = 5 (3, 5, 6, 10 m). m—month.

Consistent with published data [31], amyloid deposition was first detectable in the subiculum at an age of approximately two to three months (Figure 2). Total Aβx-42 plaque load (defined as the relative area stained with the rabbit monoclonal antibody D3E10) increased rapidly between two and six months and leveled out between six and ten months, reaching a relative area of 10.22% ± 1.86% (mean ± SD; Figure 2 and Figure 3A, Supplementary Figure S1A and Table 1).

The observed time course of Aβ1–x deposition in the subiculum (assessed with mAb82E1) was clearly different: The relative Aβ1-x positive plaque area increased rapidly between two and five months of age reaching a peak value of 17.57% ± 2.94% (mean ± SD), followed by a substantial decrease to 9.95% ± 2.24% at 10 months (Figure 3B). 

In contrast, the relative area covered by amyloid deposits consisting of N-truncated Aβ peptides starting with phenylalanine residue at position four (Aβ4-x) increased constantly from 0.41% ± 0.45% at two months to 7.6% ± 1.51% at ten months (Figure 2 and Figure 3C). 

**Figure 4:**
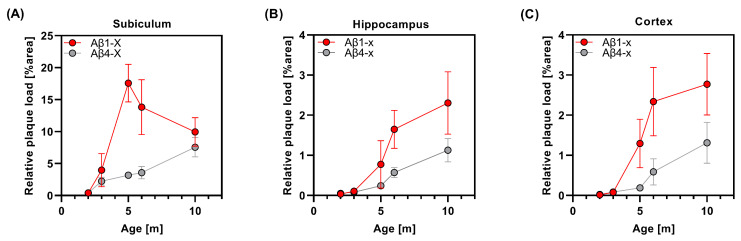
Direct comparison of Aβ1-x and Aβ4-x amyloid load during aging in the subiculum (**A**), hippocampus (**B**) and cortex (**C**) of male 5XFAD mice. m—month.

This ongoing increase in N-truncated Aβ4-x over time and the concomitant reduction in the amount of full-length Aβ species in the subiculum after five months suggest that proteolytic modifications after initial Aβ1-x deposition may play a major role in the remodeling of amyloid plaque composition in the aging 5XFAD mouse brain, at least in brain areas with a high plaque burden. A direct comparison of the time course between the deposition of Aβ1-x and Aβ4-x in the subiculum is shown in Figure 4A. 

In the hippocampus, total Aβx-42 (Figure 3D), as well as Aβ1-x (Figure 3E) and Aβ4-x (Figure 3F), showed a continuous increase over time, reaching a maximum at ten months of age. Plotting the Aβ1-x and Aβ4-x plaque load values against the age indicated a continuous increase in the curves until ten months (Figure 4B), while a flattening was observed for Aβx-42 (Supplementary Figure S1B). The time course of Aβ deposition in the cortex was similar to that in the hippocampus (Figure 3 and Figure 4 and Supplementary Figure S1C). While almost no plaques were detected in young 5XFAD mice between two and three months of age (Figure 3G–I), plaques were clearly detectable at the age of five months and showed increasing amounts up to the age of ten months.

The overall Aβx-42 plaque load in the cortex showed a continuous increase over time in this brain area (Figure 3G; Supplementary Figure S1C). This was also observed for full-length Aβ1-x, though the curve flattened between six and ten months of age (Figure 3H and Figure 4C). In good agreement with the observation in the subiculum and hippocampus, the amount of plaques consisting of N-terminally truncated Aβ4-x peptide variants showed a continuous increase between six and ten months of age (Figure 3I and Figure 4C).

**Figure 5:**
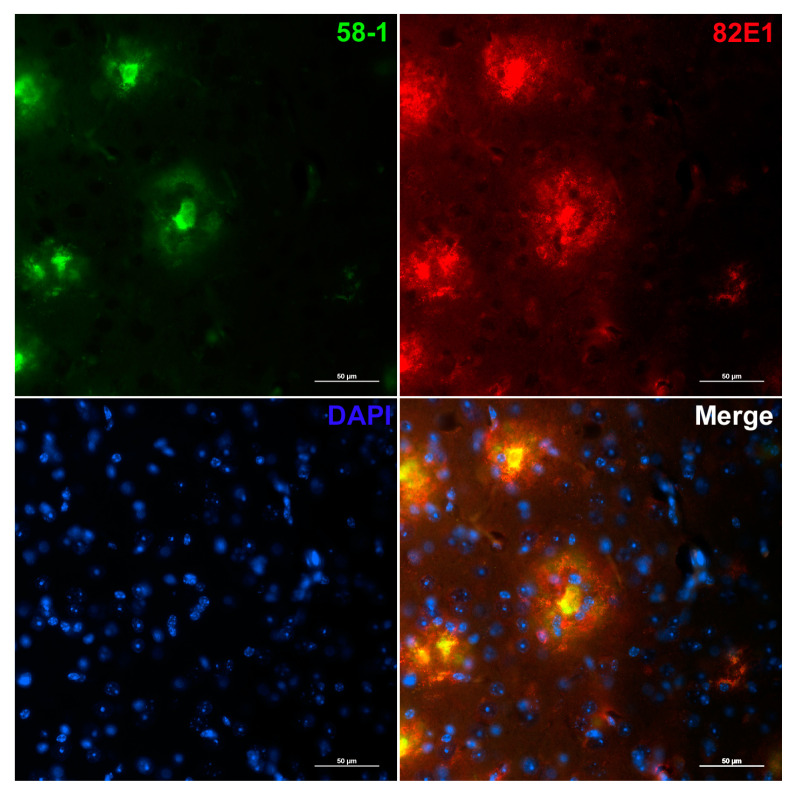
Double immunofluorescence staining with antibodies detecting Aβ1-x (82E1) and Aβ4-x (58-1) species in the cortex of a 10-month-old 5XFAD mouse. Note that Aβ4-x peptides are concentrated in the dense plaque core, while Aβ1-x peptide variants show a more diffuse and widespread distribution. Scale bar: 50 µm.

In order to further evaluate the relationship between different Aβ peptide variants in the cortex of aged 5XFAD mice, double-staining against Aβ1-x and Aβ4-x was performed. The mouse monoclonal anti-Aβ1-x antibody 82E1 was incubated together with the Aβ4-x-selective polyclonal rabbit antibody 58-1 generated by immunization with a peptide comprising amino acids 4–9 of the Aβ sequence (Supplemental Information). Visualization with fluorescent secondary antibodies revealed a clear concentration of Aβ4-x peptide species in the plaque core, while Aβ1-x species showed a more widespread diffuse distribution pattern at the plaque borders in aged 5XFAD mice (Figure 5). 

## 4. Discussion

In the present study, extracellular amyloid plaque load was quantitatively assessed in different brain regions of the widely used 5XFAD mouse model of AD in an age-dependent manner. Analyses were carried out in the cortex, the hippocampal formation and the subiculum in male animals aged two to ten months. Though the overall plaque load, as determined by staining with an antibody against the carboxy-terminus of Aβx-42 which does not discriminate between N-terminally modified isoforms, constantly increased in all brain regions, the amount of full-length Aβ1-x species in the subiculum showed a different pattern. While a time-dependent increase of Aβ1-x was also observed in the hippocampus and cortex, the subiculum, representing the area with the highest relative plaque density, showed a peak at five months, which was followed by a substantial decrease at later time points.

In recent years, several N-terminally truncated Aβ species have been identified in the human AD brain [19,20,40,41], as well as in the human APP-transgenic mouse brains [42]. Aβ variants starting with a phenylalanine residue at position 4 are among the most abundant Aβ peptide species in the human AD brain [20]. They were first discovered in amyloid plaque cores almost four decades ago. Using Edman protein sequence analysis, Masters and colleagues reported that only 10% of the Aβ peptides purified from amyloid plaque cores from AD subjects contained the intact N-terminus starting with an aspartic acid residue at position 1, while more than 60% were N-terminally truncated and started with a phenylalanine residue [3]. This result was confirmed by Miller and colleagues, who showed that a core extract preparation obtained by size-exclusion chromatography and subjected to amino-terminal sequencing yielded a sequence beginning with Phe-4 [43].

While it has been previously suggested that N-terminally truncated Aβ peptides found in amyloid plaques are rather formed directly at “β-secretase level” and not through progressive proteolysis of full-length Aβ1-40/1-42 [44], more recent research does not support such a mechanism. In good agreement with the present observation, increased formation of pyroglutamate-modified Aβ3-x (AβN3pE) appeared to occur in aged APP/PS1KI mice at the expense of full-length Aβ starting with the aspartic acid residue [45]. We recently identified an enzymatic mechanism accountable for the direct generation of Aβ4-x from its precursor APP [22]. Overexpression of the enzyme ADAMTS4 (a disintegrin and metalloprotease with thrombospondin motifs 4) leads to increased amounts of Aβ4-40 peptides in APP-transfected HEK293 cells, while 5XFAD mice lacking endogenous ADAMTS4 presented with significantly reduced Aβ4-x peptide levels. There is also evidence that full-length Aβ peptides are substrates for further proteolytical cleavage. Aβ4-x peptides can be generated in vitro from synthetic, full-length Aβ1-42 by recombinant ADAMTS4 [22] or recombinant neprilysin [46]. As most of the known Aβ-degrading proteases, such as neprilysin or insulin-degrading enzyme, belong to the family of zinc metalloproteases [47], the distribution of both the enzyme itself as well as the availability and abundance of the respective metal ion needed might play an important role for its proper activity. Zinc is present in high concentrations in the brain regions, such as the lateral amygdala or subiculum [48], which may contribute to the generation of N-terminally modified Aβ peptides. The expression of NEP and IDE has also been observed in astrocytes, especially under conditions of stress such as hypoxia [49]. Since extracellular amyloid deposition is associated with a strong neuroinflammatory response, it is likely that the levels of these enzymes are elevated, especially in brain regions with a high plaque burden. It has been further suggested that amyloid plaques in the brain can decompose from Aβ1-40/Aβ1-42 to N-terminally truncated species in a spontaneous manner by non-enzymatic processes [50].

As shown before in human AD samples and transgenic AD mouse models, Aβ4-x immunoreactivity is primarily concentrated in the core of the plaque [26,27]. Our observation of an age-dependent decrease in Aβ1-x species in the subiculum of 5XFAD mice is interesting as this is the region of initial extracellular amyloid deposition. In good agreement with our data, it has been further demonstrated that the subiculum is among the regions with the highest density of amyloid deposits in the 5XFAD mouse model [31]. It has been suggested that microglia may play a role in plaque maintenance and possibly also remodeling. Local resident microglia rapidly reacted to plaque formation and it has been demonstrated that subsets of plaques change their size over time with an increase or decrease related to the volume of associated microglia [51]. This may, at least in part, be mediated by the triggering receptor expressed on myeloid cells 2 (TREM2). In 5XFAD mice with or without TREM2 deficiency, Aβ pathology was comparable at time points around the onset of plaque deposition [52]. Interestingly, in 8-month-old animals, the abundance of the N-terminally truncated AβpE3-42 variant relative to Aβ1-42 was reduced in 5XFAD mice lacking the TREM2 receptor.

A limitation of the current study is that the overall Aβ plaque load was only assessed with an Aβ42-selective antibody. As can be seen in the subiculum, the detected amount of Aβ1-x species exceeds the levels measured with the Aβx-42 antibody. This is likely due to the fact that 5XFAD mice also accumulate substantial amounts of Aβx-40 peptides [31,32] but may also be related to differences in antibody sensitivity.

In summary, immunohistochemical analysis of brain sections from the 5XFAD mice showed a general increase in plaque burden in the subiculum, hippocampus and cortex during the first five months after birth. In the subiculum, the area with the greatest relative plaque coverage, Aβ1-x burden peaked at five months of age and decreased slightly thereafter, whereas immunoreactivity of Aβx-42 and Aβ4-x in amyloid deposits increased steadily during the first ten months of life, suggesting that there is evidence of plaque remodeling in an age-dependent fashion.

## Figures and Tables

**Table 1:** Plaque load [mean area in % ± SD] of different Aβ species in the subiculum, hippocampus, and cortex during aging. m—month.

Brain Area	2 m	3 m	5 m	6 m	10 m
Subiculum					
Aβ_x-42_	0.6 ± 0.29	2.83 ± 0.98	6.47 ± 0.59	9.79 ±1.98	10.22 ± 1.86
Aβ_1-x_	1.38 ± 0.71	3.97 ± 2.58	17.57 ± 2.94	13.81 ± 4.31	9.95 ± 2.24
Aβ_4-x_	0.41 ± 0.45	2.24 ± 0.64	3.17 ± 0.43	3.56 ± 0.97	7.56 ± 1.51
Hippocampus					
Aβ_x-42_	0.03 ± 0.013	0.1 ± 0.04	0.5 ± 0.05	1.4 ± 0.32	1.6 ± 0.28
Aβ_1-x_	0.04 ± 0.018	0.11 ± 0.06	0.76 ± 0.59	1.65 ± 0.48	2.31 ± 0.78
Aβ_4-x_	0.05 ± 0.05	0.08 ± 0.02	0.24 ± 0.07	0.57 ± 0.13	1.13 ± 0.29
Cortex					
Aβ_x-42_	0.02 ± 0.02	0.09 ± 0.06	0.56 ± 0.12	1.96 ± 0.31	2.55 ± 0.6
Aβ_1-x_	0.014 ± 0.02	0.08 ± 0.06	1.3 ± 0.61	2.34 ± 0.85	2.77 ± 0.77
Aβ_4-x_	0.03 ± 0.04	0.09 ± 0.02	0.19 ± 0.02	0.59 ± 0.33	1.31 ± 0.51

## Data Availability

Original data is available from the authors upon reasonable request.

## Supplementary Materials

The following supporting information can be downloaded at: https://www.mdpi.com/article/10.3390/life13041053/s1. Figure S1: Time-dependent accumulation of Aβx-42 in amyloid plaques in selected brain regions of male 5XFAD mice; Figure S2: Dot-blot assay; Figure S3: Aβ4-40 peptide blocking experiment. Reference [53] has cited in the Supplementary Materials.
