# Supplementary material for: Brain Region-Specific Differences in Amyloid-β Plaque Composition in 5XFAD Mice
Source: Life (Basel). 2023 Apr 20;13(4):1053. doi: 10.3390/life13041053 (PMC10145597; doi:10.3390/life13041053)
Supplement: Supplementary file 1 [file life-13-01053-s001.zip › life-2316959-supplementary.pdf]

Supplementary Materials

Brain Region-Specific Differences in Amyloid-β Plaque Composition in 5XFAD Mice

Angelika Sabine Bader, Marius-Uwe Gnädig, Merle Fricke, Luca Büschgens, Lena Josefine Berger, Hans-Wolfgang Klafki, Thomas Meyer, Olaf Jahn, Sascha Weggen and Oliver Wirths

1. Time dependent accumulation of Aβx-42 in amyloid plaques in selected brain regions of male 5XFAD mice

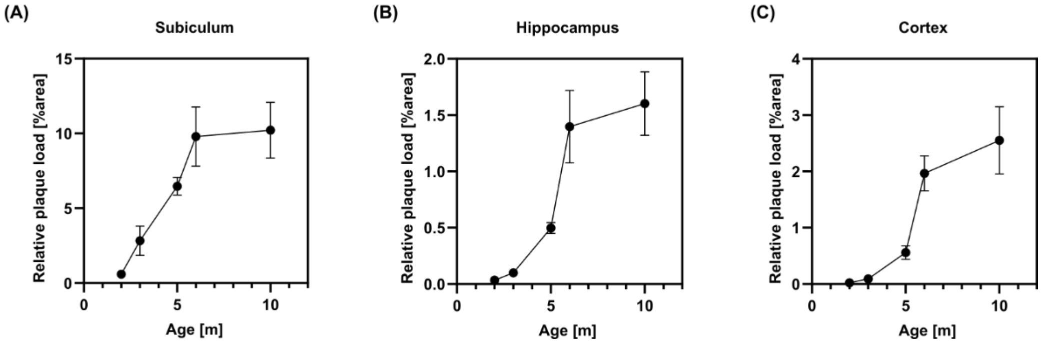

Figure S1. Time dependent accumulation of Aβx-42 in amyloid plaques in the subiculum (A), hippocampus (B) and cortex (C) of male 5xFAD mice detected with the monoclonal rabbit anti-Aβx-42 antibody D3E10 (Cell Signaling). The relative area occupied by amyloid deposits was plotted against the age of the animals. Aβx-42 deposition increased from age 2 to 6 months and levelled between 6 and 10 months of age (n = 7 (2m), n = 5 (3 - 10 months). All data are given as means ± SD).

2. Generation of A*β*4-x-specific polyclonal antibodies in rabbits

Antibodies were generated by Moravian-Biotechnology (Czech Republic) on a contract basis. In brief, two rabbits were immunized with the six-amino acids long peptide FRHDSG corresponding to residues 4 – 9 of the Aβ peptide coupled via an additional carboxy-terminal cysteine residue to keyhole limpet hemocyanin (KLH). Antisera were generated by injection of the KLH-coupled peptide emulsified with complete Freund's adjuvant for a first injection, followed by booster injections in week 3, 7 and 11. Serum was collected one week after the fourth immunization. Two rounds of affinity purification were carried out using the SulfoLink immobilization kit for proteins (Thermo Scientific). In the first round, the heptapeptide DAEFRHC corresponding to the N-terminal 6 amino acid residues of the A*β* sequence plus an additional cysteine for coupling was used for negative selection to remove antibodies detecting the intact A*β* N-terminus. The flow-through was further purified on columns carrying a peptide corresponding to amino acids 4-9 plus cysteine (FRHDSGC), to enrich antibodies selective for the phenylalanine in the N-terminal position. Successful peptide coupling was monitored with HPLC, and purified antibodies were eluted with 200 mM glycine, pH 2.5. The initial two elution fractions were pooled, concentrated with Amicon Ultracel-30 centrifugal filters with a 30 kDa cut-off (Merck Millipore) and stored in a concentration of 1 mg/ml (named 58-1) at -20°C until further use.

3. Dot-blot assay

To assess the selectivity of the polyclonal anti-Aβ4-x antibody 58-1 for different N- terminal variants of Aβ, a dot-blot immunoassay was employed. Synthetic A*β* peptides (Aβ1-40, Aβ2-40, Aβ3-40, Aβ4-40 and Aβ5-40) were spotted in a 2 µl volume on nitrocellulose membranes in amounts of 20, 100 and 250 ng. After drying, membranes

were blocked in 10% non-fat dry milk in PBS for 1 h at room temperature and incubated overnight in PBS with either the control antibody 24311 (pan-Aβ, 1:1000, [53]) or 58-1 (1:1000). On the next day, the membranes were washed three times in PBS containing 0.1% Triton X-100, followed by incubation with horseradish peroxidase (HRP)-conjugated goat anti-rabbit IgG (1:50,000 in PBS-T) for 1 h and subsequent detection with a chemiluminescent substrate (ECL Prime Western blotting detection reagent, Amersham).

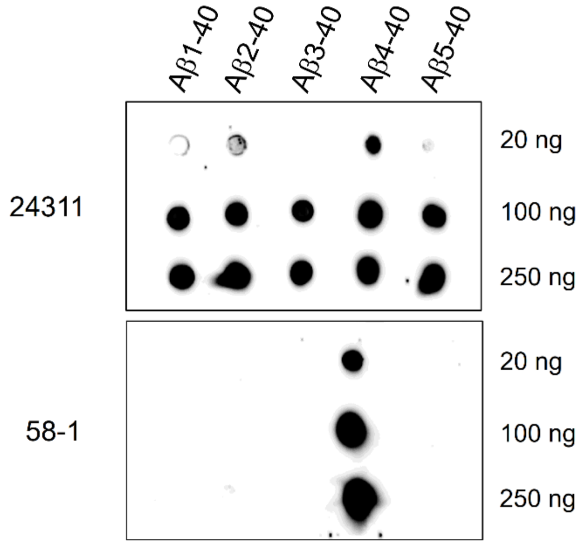

Figure S2. While the control antibody 24311 detected full-length Aβ as well as all N-terminal truncated Aβ variants, (upper panel), antibody 58-1 showed excellent selectively for Aβ4-40 (lower panel).

4. Aβ 4-40 peptide blocking of antibody 58-1

In order to further assess the selectivity of antibody 58-1, a peptide blocking experiment with synthetic Aβ4-40 peptides was performed. In brief, 2 µg of 58-1 were incubated with synthetic Aβ4-40 peptides in a 10-fold excess (20 µg) in a total volume of 1 ml PBS for 3 h at room temperature with constant rotation. The control experiment contained only 2 µg of 58-1 in 1 ml PBS without the synthetic peptides. Following incubation, the vials were centrifuged at 13,000 rpm for 5 min and the supernatant was used for overnight incubation of paraffin-embedded tissue slides of 4 µm thickness from 6-month-old 5XFAD mice. On the next day, slides were washed with 0.01 M PBS including 1% Triton X-100 and were incubated with an anti-rabbit-biotinylated antibody (Jackson ImmunoResearch, 1:200) for 1 h at 37°C. Staining was visualized using the ABC method with a Vectastain kit (Vector Laboratories, Burlingame, CA, USA) with diaminobenzidine (DAB) as chromogen and hematoxylin counterstaining.

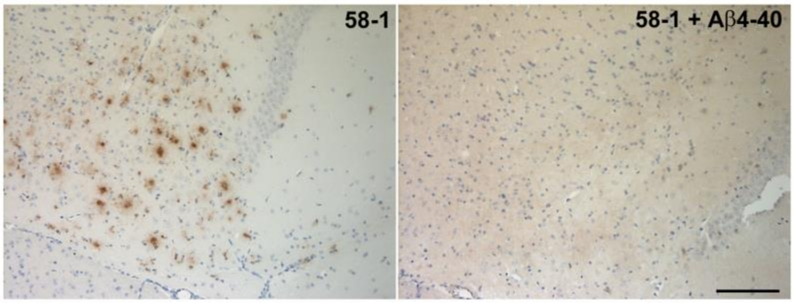

Figure S3. Immunohistochemical staining of a 6-month-old 5XFAD mouse with antibody 58-1 reveals abundant extracellular immunoreactivity in the subiculum. Pre-incubation of 58-1 with synthetic Aβ4-40 peptides entirely blocks the immunostaining (scale bar: 100 µm).
